# Preparation for a pandemic: The need for a standardised African health protocol for diseases

**DOI:** 10.4102/jphia.v16i1.735

**Published:** 2025-01-13

**Authors:** Ebenezer K. Frimpong, Mlungisi Ngcobo, Nceba Gqaleni

**Affiliations:** 1 Traditional Medicine Laboratory, School of Nursing and Public Health, College of Health Sciences, University of KwaZulu-Natal, Durban, South Africa; 2 Africa Health Research Institute, Nelson R. Mandela School of Medicine, Durban, South Africa

Dear Editor,

The novel coronavirus disease 2019 (COVID-19), which originated in China has been a threat to human lives and economic activities in both developing and developed countries.^1,2^ The negative impact of COVID-19 on economies in the respective countries is highly evident because of the loss of revenue and high unemployment rate.^3^ The World Bank report indicated that the impact of COVID-19 on the global economy increased global poverty and inequality.^4^

Generally, on the African continent, the number of active COVID-19 cases and casualties in various countries is far less compared to countries in other continents. Based on the available data as of 28 April 2024 the number of people who have acceded to the virus in the different continents is as follows: Europe (2 272 097), Americas (3 018 275), South-East Asia (808 638), Africa (175 510) and Western Pacific (420 809).^5^

One might argue that the low numbers of COVID-19 cases from the African continent could be because of underreporting or limited COVID-19 testing capabilities in the various countries. Whether the argument is true or false per the low numbers of COVID-19-related deaths (data from the various continents) established on the 28 April 2024, the information regarding the COVID-19 fatalities on the African continent could be true.^5^

Is COVID-19 here to stay? Is it going to be the last pandemic? The answer to this million-dollar question is a big ‘NO’. How prepared is Africa to face the next pandemic? To prepare adequately to face the next pandemic, the proposed interventions in the following section should be considered by the African Union (AU) member countries.

## Africa Health Research Fund

There is an urgent need to establish an African Health Research Fund (AHRF). The AU member countries are supposed to contribute a certain amount of money annually to assist with the research and development (R&D) on the African continent. It is difficult to understand why a continent that accounts for 13.5% of the world’s population contributes to less than 1% of the global research output.^6^ It is significant to point out that the countries that invest a lot in R&D have stronger economies and efficient healthcare systems. Some of the countries that invest a lot in R&D are Switzerland, the United States, Finland and Singapore.^7^ This means that the more a nation invests in R&D, the stronger its economy and vice versa.

## Africa Research Command Centre

The proposed Africa Research Command Centre (ARCC) should be positioned in one of the AU member countries. The ARCC should comprise representatives from the biomedical health professionals (BHPs), traditional health practitioners (THPs), the Department of Science, Innovation and Technology (DSIT) and legal experts involved in intellectual property (IP) and research activities. The ARCC will coordinate all the R&D-related activities on the African continent. Moreover, it will assist novel scientific research projects on the continent. Researchers having difficulties with patenting their work will also be assisted.

## Recognition and development of Indigenous Knowledge Systems

Africa’s quest to establish a standardised health protocol for diseases cannot be achieved without the development of Indigenous Knowledge Systems (IKS) in various countries. It is about time we made good use of IKS, which assisted our ancestors to overcome health challenges in the past before the advent of Orthodox Conventional Medicine (OCM). Within the past decade, there have been efforts to develop IKS on the African continent. One country that deserves commendation for championing the development of IKS is South Africa. South Africa is one of the few countries working towards the integration of both traditional medicine (TM) and OCM for health practitioners.^8^ Some of the universities in South Africa have well-established IKS centres, which are assisting a lot of IKS-related health projects across the country. More importantly, the North-West University has a faculty that offers IKS undergraduate to postgraduate degree levels.^9^ Another AU member country, Ghana, has developed a pharmacopoeia of indigenous medicinal plants.^10^

**FIGURE 1:**
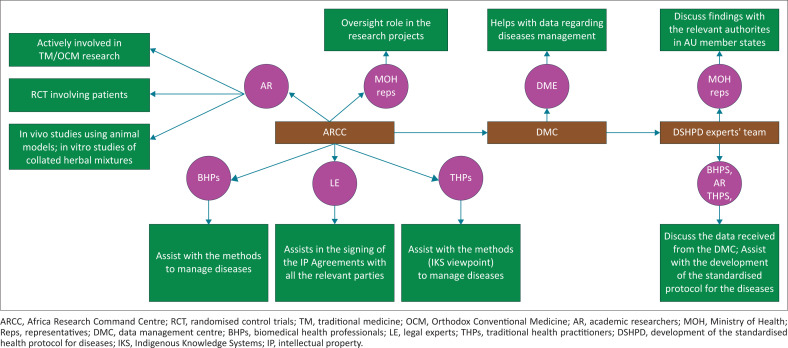
A graphical view showing the proposed guidelines for the establishment of a standardised protocol for diseases on the African continent.

To get the best out of IKS in our quest to achieve a robust healthcare system on the African continent, there should be the establishment of TM and OCM research centres in all the AU member countries. The proposed TM-OCM research centres should hire BHPs, THPs and experts actively involved in both TM and OCM research to share ideas on how to improve the health and well-being of the citizens in AU member countries. To achieve this aim, AU member countries should make all the necessary efforts to ensure that information about signs and symptoms of diseases, medicinal plants and the constituents of herbal mixtures employed by THPs in the management of diseases is well documented. Research carried out by Frimpong and Nlooto suggests that THPs are often reluctant to reveal information about the medicinal plants and constituents of the herbal mixtures in the management of diseases for fear of losing knowledge to scientific researchers who make money out of it without giving them a share of the profit.^11^ The office of DSIT in AU member countries, with the assistance of their legal representatives, must sign IP agreements with THPs to make sure that they also benefit from their hard-earned knowledge. There should be efforts for the collection and documentation of relevant information from THPs. This information obtained from the THPs about medicinal plants and the constituents of their herbal mixtures will be critical to assist scientific researchers in performing all the necessary laboratory analyses to evaluate their efficacies. Available data on the findings from the respective TM-OCM collaboration research centres (Figure 1) must be sent to ARCC for comparative analysis, which will assist in the establishment of a standardised health protocol for diseases on the continent.

The TM-OCM research centres situated in the respective AU member countries would be able to train THPs about the use of instruments such as sphygmomanometers, thermometers and glucometers on their patients who rely on their services for their primary healthcare needs. The THPs will also teach the BHPs about the need to understand the management of diseases from a cultural point of view.

## Collaboration between institutions of higher learning on the African continent

To achieve an efficient healthcare system through the establishment of a standardised health protocol for diseases within the AU member countries, R&D collaboration between institutions of higher learning on the African continent must be taken into consideration.

Institutions of higher learning in AU member countries specialising in specific areas of research should be identified. There should be well-coordinated research collaborations between students and lecturers at the various universities on the African continent. The universities on the continent that are less endowed must be assisted with facilities and the training of their faculty members by experts in a specific area of research.

## Development of a standardised health protocol for diseases

The development of a standardised health protocol to fight diseases will be made possible via research work executed by scientists in the various AU member countries with the assistance of the ARCC. Frimpong et al.^12^ reported the utilisation of similar medicinal plants by different African countries to manage headache disease on the continent. There is a need to replicate similar studies for other diseases on the continent. Findings from these studies will assist us in documenting medicinal plants via the ARCC that can be employed to manage diseases on the continent. These identified medicinal plants will be subjected to scientific evaluation to ascertain their efficacies. Data from these proposed studies will assist us in the development of a standardised protocol for the management of diseases on the continent. The standardised health protocol can be developed by using other indigenous health systems, which have undergone a similar process such as the Ayurveda standard treatment guidelines for diseases.^13^ The development of standardised protocols for diseases will assist AU member countries in preparing and fighting any pandemic in the foreseeable future.

## Conclusion

In conclusion, the possibility of an emergence of a future pandemic is inevitable. However, timely implementation of the proposed interventions enumerated in this letter will assist greatly in the mitigation of the effects of any pandemic on the African continent.
